# Promoting the Aging Process and Enhancing the Production of Antioxidant Components of Garlic through Pulsed Electric Field Treatments

**DOI:** 10.3390/antiox13030374

**Published:** 2024-03-19

**Authors:** Chao-Kai Chang, Sheng-Yen Tsai, Ming-Shiun Tsai, An-Ting Tu, Chih-Yao Hou, Kuan-Chen Cheng, Wei-Lun Zhu, Rizka Mulyani, Chang-Wei Hsieh

**Affiliations:** 1 Department of Food Science and Biotechnology, National Chung Hsing University, South District, Taichung City 402227, Taiwan; kai70219@nchu.edu.tw (C.-K.C.); g111043211@mail.nchu.edu.tw (A.-T.T.); d111043003@mail.nchu.edu.tw (W.-L.Z.); 2 Department of Medicinal Botanicals and Health Applications, Da-Yeh University, Dacun District, Changhua County 515006, Taiwan; tsaims1@mail.dyu.edu.tw; 3 Department of Seafood Science, National Kaohsiung University of Science and Technology, Nanzi District, Kaohsiung City 811532, Taiwan; 4 Department of Medical Research, China Medical University Hospital, North District, Taichung City 40428, Taiwan; kccheng@ntu.edu.tw; 5 Graduate Institute of Food Science and Technology, National Taiwan University, Daan District, Taipei City 106319, Taiwan; 6 Institute of Biotechnology, National Taiwan University, Daan District, Taipei City 106319, Taiwan; 7 Department of Optometry, Asia University, Wufeng District, Taichung City 413305, Taiwan; 8 Department of Agricultural Product Technology, Universitas Sebelas Maret, Kentingan, Surakarta 57126, Indonesia; rizka.mulyani@staff.uns.ac.id; 9 International Doctoral Program in Agriculture, National Chung Hsing University, South District, Taichung City 402227, Taiwan

**Keywords:** pulsed electric fields (PEFs), aged garlic, S-allyl-L-cysteine, γ-glutamyltransferase, principal component analysis

## Abstract

Shortening the aging duration and enhancing the functional components of garlic present significant technical challenges that need to be addressed. Thus, this study aimed to evaluate the potential role of pulsed electric field (PEF) treatment, a novel nonthermal food processing method, in promoting and enhancing the functional attributes of aged garlic. Our results showed that 2–4 kV/cm PEF pretreatment increased S-allyl cysteine (SAC), total polyphenol (TPC), and flavonoid contents (TFC) compared with un-pretreated garlic during aging. The browning and texture-softening were also significantly improved during processing time, though the latter showed no significant difference from the eighth day to the end of the aging process. The principal component analysis results showed that PEF positively affects the SAC and TFC formations without adverse effects. Among the PEF pretreatments, 3 kV/cm is the most effective in enhancing functional component production compared with the other PEF pretreatments. Therefore, PEF pretreatment is a time-saving process that promotes and enhances the functionality of aged garlic.

## 1. Introduction

In many Asian cultures, aged garlic is a healthy food due to its enhanced properties. The aging process, achieved through careful regulation of temperature and humidity, alters the texture and the functional characteristics of garlic [1]. This transformation results in a product that is different in taste and consistency compared with fresh garlic and potentially offers more substantial health benefits, especially due to an increased antioxidant content [2]. In the process of thermal processing, heat acts on the cellular structure of food, disrupting the bonds between the cell wall and polyphenolic constituents [3]. The disruption of these bonds facilitates the release of polyphenols in a free form, enhancing their bioavailability and efficacy [4]. In addition, during the heating process, the hydroxycinnamic acid derivatives of garlic form flavonoids due to the action of phenyl styrene ketone synthase (chalcone synthase, CHS), thereby enhancing the antioxidant capacity of garlic [5]. However, CHS, whose optimal reaction temperature is 30–45 °C [6], is likely inactivated by the traditional method of aged garlic at temperatures above 70 °C [7]. Therefore, destroying the internal structure of garlic during the aging process without affecting the enzymatic activities to promote the formation of free polyphenols and flavonoids is a technical challenge to improve the functionality of aged garlic.

S-allyl cysteine (SAC), the most representative functional component of garlic, is a water-soluble, highly stable sulfur-containing amino acid [8]. SAC shows the functions of anti-inflammation, nerve protection, anti-oxidation, and promotion of gastrointestinal motility [9]. SAC is produced from the reaction of catalyzing glutamyl-S-allyl-L-cysteine (GSAC) by γ-glutamyl transpeptidase (γ-GTP). However, GSAC and γ-GTP are usually blocked by the garlic cell wall, resulting in a limited abundance of SAC in garlic. The traditional aging process of garlic uses a high temperature above 70 °C to cause irreversible damage to the cellular structure and then increases the reaction rate by γ-GTP to generate SAC [8,10]. Therefore, it is vital to establish a processing method that can moderately destroy the garlic structure without affecting the enzymatic activities during aging.

Fructan, the main polysaccharide in garlic [11,12], promotes gastrointestinal motility, resists inflammation [13], and improves gastric ulcers [14]. During the aging process of garlic at high temperatures (70–90 °C), the reducing sugar produced by fructan degradation causes a brown appearance [15] and a sweet flavor of aged garlic [16,17]. A previous study shows that garlic’s endogenous fructan exohydrolase (FEH) degrades fructan into sucrose and fructose at 45 °C [18]. In addition, Bae et al. [19] show that garlic aging at 40 °C benefits from SAC formation. However, the browning rate and antioxidant capacity are much lower than those of garlic aged at 85 °C.

The above literature indicates that the high temperature (70–90 °C) in the traditional aged garlic process promotes the Maillard reaction, SAC formation, and the release of free polyphenols. In addition, the high temperature in the traditional aging process degrades the structure of matured garlic. Nevertheless, some studies have also clearly mentioned that these temperatures limit the enzymatic activities that generate SAC and free flavonoids, thereby limiting the functionality of aged garlic [18].

In recent years, nonthermal pretreatment has garnered considerable interest as a substitute for high temperatures in the traditional aged garlic process. Our previous study shows that nonthermal pretreatment techniques such as freezing ultrasound-assisted and high hydrostatic pressure are beneficial to enzymatic reactions during garlic aging, thereby improving the functionality of aged garlic [20,21]. Moreover, a previous study showed that electric field pretreatments such as constant electric field and pulsed electric field could be applied to affect the quality of black garlic through the effect of electric fields, with the latter showing remarkable activity to affect the configuration of enzymes, thereby interfering enzymatic activity [22]. Pulsed electric field (PEF) is a nonthermal emerging food processing technology generated by short pulse waves and various electric field strengths [23,24,25]. It is worth noting that PEF treatment can be used to assist in drying, frying, and fermentation by destroying cellular membrane structure and improving cell membrane permeability, as well as enabling the preservation of nutrients and minimizing flavor degradation during processing compared with other nonthermal treatments [24,26,27,28]. However, the effect of PEF intensities on increasing the functional attributes and enabling the browning and texture of aged garlic is still unknown. Therefore, this study investigated the effects of varying PEF intensities on functional components, such as polyphenols, flavonoids, fructans, and SAC, during the aging process of garlic. In addition, a comprehensive evaluation was conducted to determine whether PEF facilitated the browning phenomenon and texture softening of aged garlic. This study aimed to comprehensively evaluate and elucidate the potential role of PEF pretreatment in promoting garlic aging and enhancing the functional attributes of aged garlic. Furthermore, this study highlights the future potential of PEF pretreatment in expediting aging processes and enhancing the functional properties of aging/fermented food.

## 2. Materials and Methods

### 2.1. Plant Materials and Treatment

The garlic (*Allium sativum*) used in this study was cultivated in Huwei Township, Yunlin County, Taiwan, provided by the Xiluo Agricultural Products Market Co., Ltd., Yunlin, Taiwan, ensuring it was mechanically damage-free. The PEF equipment used in this experiment contains a PEF generator (PG403751, Youshang Technical Corp., Kaohsiung, Taiwan) and a direct current supply (DSP-450-03-4HD, Chin Hong Electronic CO., LTD, Taipei, Taiwan). The treatment chamber consisted of two copper plate electrodes (250 × 350 mm^2^) with a thickness of 1 mm. The electrode space was fixed to 55 mm. The strength of pulsed electric field treatment referred to our previous research [29]. Each garlic bulb (300 g) was separated into cloves. Then, the outer skin of the cloves was carefully peeled off to avoid enzyme activation. A total of 60 cloves, each weighing approximately 3 ± 0.5 g, were subsequently positioned on copper electrodes for PEF treatment. The garlic cloves were directly exposed to electric field strengths of 2, 3, and 4 kV/cm (frequency: 50 Hz; pulse width: 2000 μs; and duty cycle: 0.1) for 30 s at 25 °C according to our previous study [30].

Following PEF treatment, the garlic samples underwent an aging process at 40 °C and 85% relative humidity (RH) within a climate chamber (HPP110eco, Memmert GmbH + Co. KG, Schwabach, Germany) for ten days. Conversely, the experiments designated for the initial day (day 0) were conducted immediately post-PEF processing. The remaining samples were frozen immediately at −80 °C in preparation for freeze-drying, which preceded the analysis for fructan, TPC, TFC, and SAC content. Aged garlic at 40 °C and 85% RH without PEF pretreatment served as a control.

### 2.2. Appearance and Browning Degree

The appearance of different samples was observed and photographed during storage. The color changes in the samples were determined using a colorimeter (TC-8600A, Denshoku, Tokyo, Japan). The ΔE and browning index (BI) [31] were calculated using Equations (1)–(3). The color of the samples was divided into *L*, *a*, and *b,* representing white/black, red/green, and yellow/blue, respectively. *L*_0_, *a*_0_, and *b*_0_ represent the colors of fresh garlic, while *L*, *a*, and *b* represent the colors of PEF-treated samples [7].

(1) $$△E=\sqrt{{\left(L-L_{0}\right)}^{2}+{\left(a-a_{0}\right)}^{2}+{\left(b-b_{0}\right)}^{2}}$$

(2) $$\mathrm{BI}=\frac{100\;(X-0.31)}{0.172}$$

(3) $$X=\frac{a+1.75L}{5.645L+a-3.012b}$$

### 2.3. Texture Measurement

The hardness of garlic was assessed using the method of Kim et al. [32] with some modifications. Textural characteristics were analyzed in garlic samples conforming to dimensions of 30 ± 2 mm in length and 18 ± 2 mm in height. A texture analyzer (Compac-100II, Sun Science Co., Ltd., Tokyo, Japan) was employed to measure hardness. Each sample underwent compression until achieving 50% deformation relative to its original height, utilizing a cylindrical probe with a diameter of 10 mm at a compression rate of 10 mm/s. The results were quantified in Newtons (N).

### 2.4. Fructan Content

The fructan content was quantified utilizing the Megazyme Fructan Assay Kit (K-FRUC, Neogen, Lansing, MI, United States). The garlic samples were first lyophilized using a freeze-drying process. Then, 0.10 g of garlic sample was homogenized in 100 mL of deionized water and stirred for 15 min at 80 °C. Subsequently, 0.2 mL of this solution was combined with 0.2 mL of a sucrase/amylase solution, and the mixture was incubated for 30 min at 40 °C to reduce sugars to sugar alcohols. Subsequently, 0.2 mL of fructanase solution was added, and the mixture was further incubated for 20 min at 40 °C to hydrolyze fructans into fructose and glucose. Finally, the resultant solution was treated with a hydroxybenzoic acid hydrazide reagent, and absorbance was measured at 410 nm to quantify the fructan content [11]. Fructan content was calculated using Equation (4) as follows:

(4) $$FC\;(mg/g)=∆A\times \frac{F}{W}\times D\times 61.9$$

where ΔA: sample absorbance − sample blank absorbance (both read against the reagent blank);

*F*: factor to convert absorbance values to μg of D-fructose = (54.5 μg D-fructose)/(absorbance for 54.5 μg D-fructose);

*W*: weight (mg) of sample extracted;

*D*: dilution fold of the sample extract.

### 2.5. FEH Activity

The FEH activity analysis referred to a previous study [18]. First, a 15 ± 0.5 g sample was added to 30 mL acetate buffer (50 mM, pH 5.0) and mixed for 30 min. The homogenate was filtered through a cheesecloth before the filtrate was centrifuged at 4000 rpm for 10 min. The supernatant (0.2 mL) was then mixed with 0.8 mL of a 3% fructooligosaccharide solution (≥90% purity, Sigma-Aldrich, St. Louis, Saint Louis, MO, United States) to assess the breakdown of fructooligosaccharides into reducing sugars using the dinitrosalicylic acid (DNS) colorimetric method. One unit of enzyme activity was defined as the amount of enzyme that liberates 1 μmoL of fructose per 1 h under the reaction conditions. The specific activity is expressed in μmol × h^−1^ per g of raw material [33]. The was calculated using Equation (5) as follows:

(5) $$E\;(U/g)=\frac{m_{2}-m_{1}}{t\;\times \;m}$$

where E (U/g) is the enzyme activity; m_1_ and m_2_ are the reducing sugar content (mg) before and after fructan hydrolysis, respectively; t is the reaction time (h); and m is the mass of garlic used in the measurement (g).

### 2.6. TPC and TFC

#### 2.6.1. Extraction of Polyphenols

The method described by the International Organization for Standardization (ISO) 14502-1 was used [34]. The garlic samples were first lyophilized using a freeze-drying process. Each sample, weighing 0.2 g, was treated with 5 mL of 70% methanol and heated at 70 °C for 10 min. Following cooling to room temperature, the mixture was centrifuged at 7840 g for 10 min. The supernatant was carefully transferred into a graduated conical tube. This extraction process was performed a total of three times. The combined extracts were then adjusted to a final volume of 10 mL using cold 70% methanol. Subsequently, 1 mL of this extract was diluted to 5 mL with water.

#### 2.6.2. Determination of TPC

The total polyphenol content (TPC) analysis refers to a previous research study [5]. The sample solution (1.0 mL) was mixed with Folin–Ciocalteu reagent (5.0 mL, 1/10 diluted) (AppliChem GmbH, Darmstadt, Germany) and 4.0 mL of a sodium carbonate solution (7.5%, *w*/*v*). The resulting solution was incubated at room temperature for 60 min. The absorbance value was measured by a spectrophotometer at wavelength of 760 nm (Multiskan SkyHigh, Thermo Fisher Scientific, Waltham, WA, United States). The TPC in the sample (mg/g dry weight, dw) was expressed in gallic acid (99.5%, Scharlau, Barcelona, Spain) equivalent (GAE) as the relative content of free polyphenols.

#### 2.6.3. Determination of TFC

The total flavonoid content (TFC) analysis was conducted based on previous research [5]. The garlic was homogenized with 1.5 mL 95% ethanol extract for 2 h. After the centrifugation (18,000 g, 15 min), 0.1 mL 10% AlCl_3_, 0.1 mL 1 M potassium acetate (CH_3_COOK), and 2.8 mL deionized water were mixed, and the absorbance at 415 nm was measured with a spectrophotometer. The TFC in the sample (mg/g dw) was expressed in Quercetin Equivalence (QE).

### 2.7. Electrolyte Leakage (EI)

The EI was determined using the method reported by Kaya et al. [35] with some modifications. The garlic samples (2 ± 0.05 g) were washed with distilled water, wiped to remove surface moisture, and put into a 50 mL beaker with 20 mL of deionized water. The electrical conductivity of the suspending solution (C_1_) was measured with a conductivity meter (WA-2017SD, Tun-Hwa Electronic Material Co., Ltd., Taichung, Taiwan) for 180 min at 25 °C. The samples were boiled for 30 min and cooled to 25 °C, and then a final conductivity measurement was taken (C_2_). The EI was calculated using Equation (6) as follows:

(6) $$\mathrm{EI}\;(\%)=(\frac{C_{1}}{C_{2}})\;\times \;100\%$$

### 2.8. γ-GTP Activity

The γ-GTP activity analysis method was based on a previous study [8]. The garlic (10 ± 0.5 g) was homogenized with 20 mL of 50 mM Tris-HCl buffer (pH 8.0). The mixture was centrifuged at 15,000× *g* at 4 °C for 15 min. Afterward, the supernatant (3 mL) was mixed with 1 mL of 40 mM methionine solution and 1 mL of 2.5 mM L-γ-glutamyl-p-nitroaniline monohydrate. The solution was incubated at 37 °C for 30 min and then boiled for 10 min to terminate the reaction. The absorbance of the solution was determined at 410 nm using a spectrophotometer. One unit of γ-GTP activity was defined as the amount of enzyme producing 1 μmol p-nitroaniline in 1 min from 4-nitroaniline. The γ-GTP activity of garlic was calculated using Equation (7).

(7) $$E\;(U/g)=\frac{C\;\times \;V\;\times \;n}{t\;\times \;m}$$

where E (U/g) is the enzyme activity; C is the concentration of 4-nitroaniline in the reaction solution (μmol/mL); V is the volume of the reaction solution; n is the volume ratio of total enzyme solution to used enzyme solution; t is the reaction time (min); and m is the mass of garlic used in the measurement (g).

### 2.9. SAC Content

The analysis of SAC content was carried out according to previous studies [8,20,21]. The garlic samples were first lyophilized using a freeze-drying process. Subsequently, the solution was heated in boiling water for 15 min, followed by ultrasound treatment for 30 min. The treated solution was then passed through 0.22 μm syringe filters. The filtered samples were analyzed using high-performance liquid chromatography (HPLC) with a UV–visible detector (L-7400, Hitachi, Tokyo, Japan). Separation of the sample employed a C18 column; the C18 column (250 mm × 4.6 mm, ID: 5 μm; Kanto Chemical, Tokyo, Japan) was used at a flow rate of 0.2 mL/min. The mobile phase consisted of distilled water (solvent A) and methanol (solvent B). The elution gradients were 0–5.5 min, 97% A; 5.5–8 min, 97–88% A; and 8–10 min, 88–80% A, 10–30 min, 80% A and 30–50 min, 80–97% A. The sample injection volume was 20 μL, and the detection wavelength was 210 nm.

### 2.10. Statistical Analysis

XLSTAT 2019 statistical software (Lumivero, Denver, CO, United States) was used to conduct principal component analysis to explore the relationship among treatments of different electric field strengths, aging indexes, and functional components of garlic.

Excel software 2019 (Microsoft, Redmond, Washington, United States) was used to conduct heatmap analysis to explore the changing trends in indicator enzyme activity and functional components in garlic aging under different electric field strength treatments and different maturation days. The codes for each group are C: control; P: PEF pretreatment; and D: heat treatment time of garlic aging. The number after P indicates the electric field strength (kV/cm) of PEF pretreatment, and the number after D indicates the time (day) of garlic aging. The green color represents high expression, and the blue color represents low expression in the heat map.

The results were analyzed using one-way ANOVA with IBM SPSS Statistics 20 software. Significant differences were determined using Duncan’s multiple range tests at *p* < 0.05. Data are represented as means ± standard deviations for four biological replicates.

## 3. Results and Discussion

### 3.1. Effects of PEF Pretreatment on Garlic’s Appearance, Browning Index (BI), and Relative Leakage (EI) during Aging

**Figure 1:**
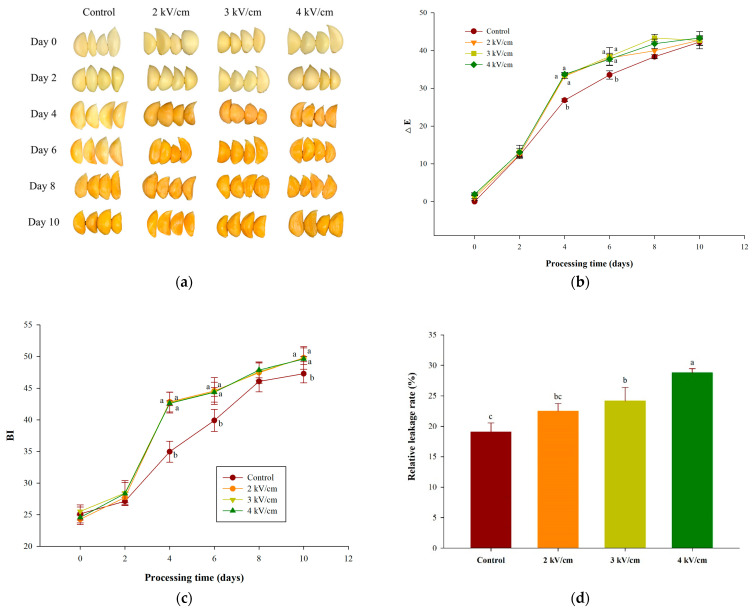
Effect of PEF pretreatments on the (**a**) appearance, (**b**) ΔE, (**c**) BI, and (**d**) relative leakage rate of garlic during aging at 40 °C for 10 days. Data are represented as means ± standard deviations for four biological replicates. Different letters show significant differences (*p* < 0.05) for each sampling date among treatments.

The effect of the PEF pretreatment on the garlic’s appearance, BI, and EI of during the aging process are shown in Figure 1. The results showed that after the PEF pretreatment, the garlic color turned brown significantly on the fourth day, while the control turned brown on the eighth day (Figure 1a). Figure 1b confirms that the ΔE values of the 2, 3, and 4 kV/cm pretreatment groups were significantly different from those of the control group after 4–6 days (*p* < 0.05). After eight days, all the ΔE values were increased to around 40 and had no significant differences. The result from Figure 1c shows that the BI values of all the PEF pretreatment groups were around 43 on the fourth day, and the BI values showed significant differences compared with the control group (35) (*p* < 0.05). The BI values of the PEF-pretreated groups on the sixth day were significantly different from those of the control group. However, the BI values between the control and PEF-pretreated groups were not significantly different from day 8 until the end of the aging process.

Our result is in accordance with a previous study [21]; the ΔE values of garlic are between 40 and 45 after pretreatment with 200 MPa high hydrostatic pressure (HHP) for 5–15 min and aged at 40 °C and 85% RH for 6 days. The results of this study show that 2–4 kV/cm PEF pretreatments for 30 s achieve a similar effect to that of HHP for 5–15 min. The other study also points out that the ΔE values of aged garlic at 45 °C after freezing at −18 °C for 30 h exceed 40 on day 4 [36].

The EI value indicates tissue and cellular membrane integrity [37]. The changes in conductivity can also indicate PEF-induced disruption of plant materials [38]. Figure 1d shows that garlic pretreated with 3 and 4 kV/cm PEF exhibited significant increases in the relative leakage rates compared with the control group, and the rates increased as the electric field strength of PEF increased. Garlic pretreated with 4 kV/cm PEF showed the highest relative leakage rate, 28.79% ± 0.68%, among all the experimental groups. PEF pretreatment causes pore formations in the cell membranes, but these formations may be reversible or irreversible depending on the PEF pretreated conditions [39]. When the PEF pretreatment forms membrane pores, intracellular ions and charged particles are released into the surrounding medium, increasing conductivity [40]. Therefore, we speculate that the PEF pretreatments destroyed garlic tissue and cell structures, significantly increasing the ΔE, BI, and EI values and accelerating garlic browning during aging.

### 3.2. Effects of PEF Pretreatments on TPC and TFC of Garlic during Aging

**Figure 2:**
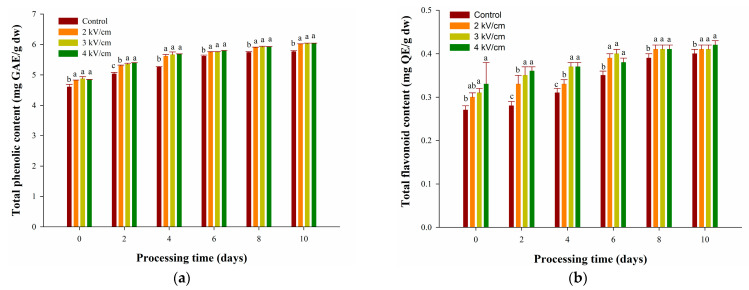
Effects of PEF pretreatments on (**a**) TPC and (**b**) TFC of garlic during aging at 40 °C for 10 days. Data are represented as means ± standard deviations for four biological replicates. Different letters show significant differences (*p* < 0.05) for each sampling date among treatments.

In order to analyze the effect of PEF pretreatment on the phenolic and flavonoid compounds of garlic during the aging process in 10 days, the TPC and TFC were analyzed. Figure 2a shows that 2, 3, and 4 kV/cm PEF pretreatments can significantly and consistently increase the TPC of garlic during the 10-day aging process compared with the control group at the same time (*p* < 0.05). In addition, the increasing TPC content of the PEF pretreatment group was consistently higher than the control group with the prolonged duration of the garlic aging process. However, the elevation of electric field intensity did not increase the TPC of garlic, as no statistically significant difference was seen among the several groups subjected to PEF pretreatment. This phenomenon resulted from the slight difference in cell permeability introduced by low electric field PEF pretreatments [41]. The study conducted by Niu et al. [42] also demonstrates that applying electric fields of 2–4 kV/cm did not yield significantly different phenolic compounds. In addition, the relatively short PEF pretreatment duration (30 s) combined with the limited capacity to degrade garlic structure at an aging temperature of 40 °C resulted in restrained increases in the TPC value during aging, as shown in the control group.

After PEF treatments, the TPC value of garlic increased from 4.8 mg GAE/g on day 0 to 6.0 mg GAE/g on day 10. On the other hand, the TPC value of the control group only rose from 4.6 mg GAE/g to 5.7 mg GAE/g, and this increase was lower compared with the groups pretreated with PEF. According to a previous study, continuous treatment of garlic at 60–90 °C, 50–100% RH disrupted the cell structure of garlic and promoted the release of conjugated phenols from garlic tissue, resulting in the formation of detectable free phenols [5]. Our study also confirmed that the 2, 3, and 4 kV/cm PEF pretreatments might lead to the release of free phenols in garlic by destroying its structure.

The previous study pointed out that PEF can inhibit the activity of polyphenoloxidase (PPO) by 80% at 1000 Hz and 5 μs at 25 kV/cm [43]. However, this study only used 2–4 kV/cm, 50 Hz, and 2000 μs. Relevant studies confirmed that lower PEF could not significantly inhibit the activity of PPO [44]. This finding elucidates that the effect of 2–4 kV/cm PEF on garlic enhanced the release of free polyphenols, but the increase in TPC was not significant.

According to Figure 2b, following PEF pretreatments of 2, 3, and 4 kV/cm, the TFCs of garlic were 0.30, 0.31, and 0.33 mg QE/g, respectively, on day 0, which were significantly higher than the control (0.27 mg QE/g) (*p* < 0.05). The TFC of the PEF pretreated and control groups increased until the eighth day, and there were no significant changes in TFC content from the eighth to the tenth day. González-Ramírez et al. [45] used 55 °C and different heating times to control different water activities (0.1–0.3 Aw) and could not increase the content of TFC in garlic. According to a study by Kim, Kang, and Gweon [5], subjecting garlic to aging conditions of 60–90 °C and 50–100% RH resulted in a notable rise in total TFC. The observed increases in TFC are mainly attributed to catechin and epicatechin, whereas quercitrin does not exhibit a significant increase under these conditions. Therefore, the effects of PEF and aging processes on different polyphenolic compounds in garlic require further study.

Figure 1a and Figure 2a demonstrate that PEF pretreatments could enhance the levels of TPC and promote garlic browning during aging. Previous studies showed that after high-pressure treatment, the discoloration of garlic during aging at 45 °C is caused by the transformation of the organic sulfur compound 1-propenyl cysteine sulfoxide (1-PeCSO) [21]. 1-PeCSO converts to thiosulfinates catalyzed by alliinase after the destruction of the garlic structure [46]. However, considering that 2–4 kV/cm is insufficient to inhibit the activity of PPO significantly, it is difficult to accurately determine at this stage whether PEF promotes garlic browning through PPO or 1-PeCSO or whether both contribute.

### 3.3. Effects of PEF Pretreatments on Texture and Fructan Content of Garlic during Aging

**Figure 3:**
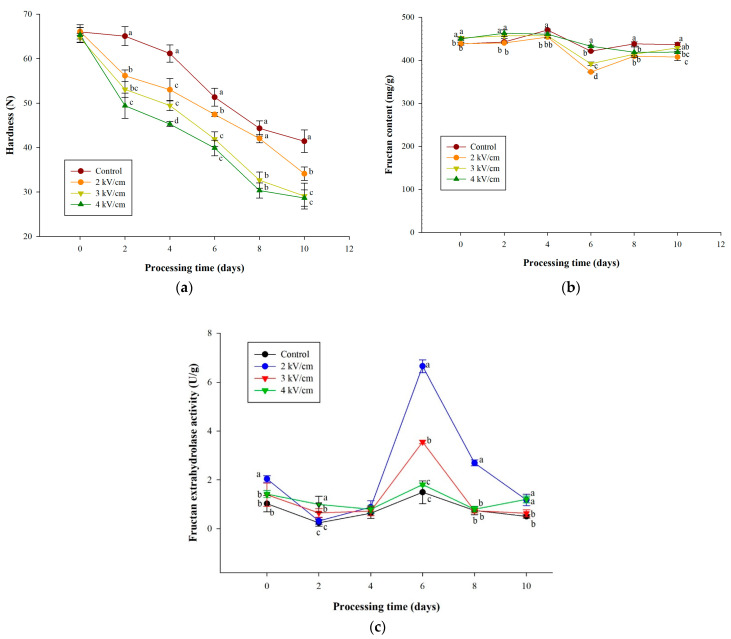
Effect of PEF pretreatment on the (**a**) hardness, (**b**) fructan content, and (**c**) FEH activity of garlic during aging at 40 °C for 10 days. Data are represented as means ± standard deviations for four biological replicates. Different letters show significant differences (*p* < 0.05) for each sampling date among treatments.

The hardness, FEH activity, and fructan content were examined to confirm the effect of PEF pretreatment on the texture and fructan content of garlic during aging (Figure 3). Figure 3a illustrates that the garlic hardness losses during the aging process for ten days in the PEF pretreatment groups were significantly higher than in the control group. The fructan content initially decreased by the sixth day, followed by a slight increase. However, the overarching trend indicated a decrease in fructan levels over time. In addition, the change in fructan was not as significant as the hardness.

Figure 3c depicts fructan extrahydrolase (FEH) activity during aging. It was shown that there was a significant increase in FEH activities of the PEF pretreated groups on the sixth day compared with the control group. During aging, as garlic’s fructan levels decreased, especially on the sixth day, the activity of fructan exohydrolase (FEH) increased, as shown in the data from Figure 3b,c. In this study, the 2 and 3 kV/cm PEF pretreated groups showed significant increases in FEH activities from 0.89 U/g to 6.65 U/g and 0.72 to 3.5 U/g, respectively, during the fourth to sixth days of aging. In contrast, the 4 kV/cm PEF pretreated group only increased from 0.79 U/g to 1.80 U/g, similar to the control group (0.63 U/g to 1.87 U/g).

Fructan accounts for approximately 23% (*w*/*w*) of fresh garlic and 75% (*w*/*w*) of dried garlic, playing a significant role in the quality and texture of garlic-based products [47]. Li et al. [48] pointed out that although high-pressure treatment will inhibit the activity of FEH, the structural destruction of garlic caused by high pressure increases the frequency of contact between the substrate (polysaccharide) and FEH, thereby accelerating the softening of the texture of garlic during subsequent aging [48]. It has been established that PEF treatment significantly impacts metabolic activities, principally initiating oxidative stress [49]. Concurrent studies have demonstrated that low-temperature stress triggers the induction of FEH gene expression in chicory roots and burdock [50,51]. Similarly, Bian et al. [52] observed a pattern of initial increase followed by a decrease in the expression of FEH genes under low-temperature conditions, which aligns with the findings of this research. The result suggests that the stress from PEF treatment may lead to changes in FEH activity.

In this study, we observed an initial decrease followed by an increase in fructan levels. Related research has elucidated that drought stress precipitates alterations in metabolic processes, signifying a critical biological response in plants to abiotic stress [53]. Such conditions facilitate the accumulation of carbohydrates, particularly affecting carbohydrate metabolism pathways in garlic. A preceding study reported that garlic mitigates oxidative damage from drought stress primarily through the induction of fructan synthesis and catabolism, with noted fluctuations in fructan levels initially increasing and subsequently decreasing [54]. The degradation and synthesis of fructans are related to FEH, 1-fructosyltransferase (1-FFT), and sucrose 1-fructosyltransferase (1-SST). Studies have shown that when garlic sprouts, the FEH enzyme activates to degrade fructans for energy metabolism, while 1-FFT is stimulated under drought and cold conditions. Simultaneously, 1-SST initiates fructan synthesis to resist cell membrane damage [55]. Therefore, the fluctuations in fructan content observed in this study may result from stress induced by PEF processing. Nonetheless, the mechanisms related to fructan content change are intricately linked to complex metabolic processes. Further exploration to understand this mechanism is critically important.

Despite the fructan content exhibiting only a slight decrease over 10 days of the aging process, the hardness of the product consistently decreased. Complementary research has elucidated that the thermal process contributes to a decrease in the hardness of garlic through membrane disruption and the depolymerization of pectic polysaccharides [56,57]. Other research has also indicated that PEF treatment may facilitate cell membrane permeabilization through electroporation, compromising the integrity of the membrane and the cell wall [58]. Therefore, the reduction in garlic hardness in this study is attributed to the synergistic effects of heat and PEF processing.

### 3.4. Effects of PEF Pretreatments on SAC Content of Garlic during Aging

**Figure 4:**
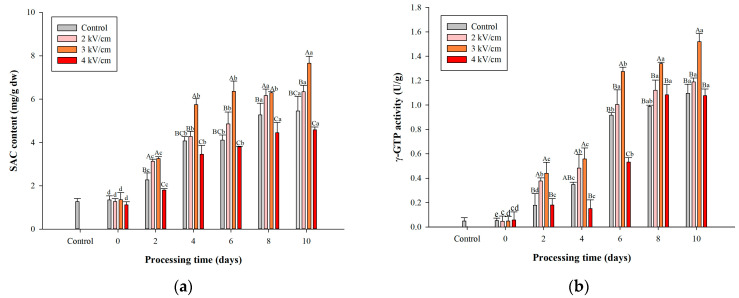
Effects of PEF pretreatments on (**a**) SAC content and (**b**) γ-GTP activity of garlic during aging at 40 °C for 10 days. Data are represented as means ± standard deviations for four biological replicates. ^a–e^ Means followed by different lowercase in the same treatment are significantly different at *p* < 0.05. ^A–C^ Means followed by different uppercases in the same processing time are significantly different at *p* < 0.05.

SAC is crucial for aged garlic’s antioxidant and other physiological functions [59,60]. Thus, increasing the content of SAC in aged garlic can enhance its nutritional value. γ-GTP is a critical enzyme that affects the formation of SAC during garlic aging [61]. Figure 4a shows that the 2 and 3 kV/cm PEF pretreatments have no adverse effect on the SAC content on day 0, but the 4 kV/cm pretreatment significantly reduces the SAC content of garlic on day 0 compared with the control group (*p* < 0.05). During the garlic aging process, the SAC content of all experimental groups increased with the aging time. The groups pretreated with 2 and 3 kV/cm PEF consistently exhibited increased SAC content compared with the control group at each aging time point. Furthermore, the 3 kV/cm PEF pretreatment group consistently had the greatest SAC content. However, for each aging time point, the 4 kV/cm PEF pretreated group consistently had the lowest SAC content among the experimental groups. These SAC content results indicated that PEF pretreatments can promote the formation of SAC during the aging process of garlic, except when the electric field intensity reaches 4 kV/cm.

Figure 4b shows that the γ-GTP activity increased as the aging time increased in all experimental groups but reached a plateau for all PEF-treated groups after day 8. The 2 kV/cm PEF-pretreated group showed a significant enhancement in the γ-GTP activity from aging day 2 to day 8, while the 3 kV/cm PEF-pretreated group demonstrated a significant increase in the γ-GTP activity from aging day 2 to day 10, compared with the control group (*p* < 0.05). Moreover, the γ-GTP activity of all pretreated samples increased by 9.8-fold to 31.0-fold compared with the control group. Among all experimental groups at the same aging time point, the γ-GTP activity was consistently higher in the group that underwent PEF pretreatment at 3 kV/cm. Furthermore, this group showed the highest γ-GTP activity, 1.52 ± 0.01 U/g, at the end of garlic aging. However, the γ-GTP activity in the 4 kV/cm PEF-pretreated group was the lowest among all the PEF-pretreated groups at the same aging time. These results (Figure 4a,b) exhibited similar trends, indicating that the experimental group had high SAC content with high γ-GTP activity.

PEF pretreatment can enhance enzyme activity such as γ-GTP, but excessive electric field causes enzyme inhibition [62]. The enhancing effect of PEF can be attributed to the generation of new active sites, alterations in the size of existing sites, and structural modifications in enzymes [63]. Therefore, the increase in substrate affinity for an enzyme leads to an acceleration of the reaction rate. A previous study reported that HHP pretreatments can enhance γ-GTP activity by 1.20 to 1.45 folds by disrupting the cell structure in garlic [20]. Our findings align with prior research indicating that PEF pretreatments can enhance γ-GTP activity via structural disruption. Previous studies have documented PEF-induced enzyme inactivation in substrates such as soymilk and egg whites by disrupting secondary and tertiary enzyme structures [64]. However, it is noteworthy that γ-GTP activity exhibited a significant reduction only on days 2 and 4 during the aging of garlic after applying a 4 kV/cm PEF pretreatment.

Following the 2 and 3 kV/cm PEF pretreatments, the SAC content increased during garlic aging (Figure 4a). The 3 kV/cm PEF electric field intensity was optimal for promoting SAC synthesis, given that the SAC content was highest at the same aging time point. In addition, it significantly increased SAC content to 6.0-fold during garlic aging for 10 days compared with the control group (3.8-fold). Furthermore, the 3 kV/cm PEF-pretreated group had the highest γ-GTP activity (7.66 ± 0.32 mg/g dw) on day 10 for garlic aging. Previous studies showed that HHP, freezing, and ultrasound pretreatments enhance the γ-GTP activity and SAC content in garlic due to the disruption of cellular structure [20,21]. In addition, using PEF in orange juice led to notable improvements in membrane permeabilities. PEF treatment also promotes the release of pectin methylesterase, which reacts with the matrix pectin. Therefore, PEF treatment increases the degradation rate of pectin [65]. In our study, the PEF pretreatments significantly affected the tissue structure of garlic, increased γ-GTP activity during aging, and promoted SAC biosynthesis during aging. However, the PEF 4 kV/cm pretreatment had a comparatively reduced SAC content than the other treatments during the aging process. This result correlated to the inactivation of γ-GTP in the 4 kV/cm treatment (Figure 4b). γ-GTP is a crucial enzyme that affects SAC formation. Consequently, the deactivation of γ-GTP at the 4 kV/cm treatment reduced SAC content during aging [39].

### 3.5. Analysis of the Relationship between PEF Pretreatments and Composition Changes during Garlic Aging

Principal component analysis was conducted to obtain a correlation between PEF treatments and composition changes during the aging process in garlic. The results indicate the factor loading value of each indicator. A positive loading value indicates a positive correlation between variable groups, while a negative value signifies a negative correlation. A factor loading value greater than 0.7 denotes a high correlation with the variable groups. Values between 0.5 and 0.7 suggest a moderate correlation, whereas a loading value less than 0.5 indicates a modest correlation with the variable groups [66]. This study used principal components (PCs) with eigenvalues >1 for analysis. Among the nine PCs, the variabilities in PC1 (74.47%) and PC2 (16.30%) account for the highest correlations and explain 90.77% of the variation. This result means the trends in each indicator after the PEF pretreatment during aging have a high correlation or consistency (Table 1). PC1 showed a high positive correlation with γ-GTP (0.951), SAC (0.948), TPC (0.962), TFC (0.940), ΔE (0.947), and BI (0.904), while PC2 demonstrated a high positive correlation with FEH (0.911). In contrast, PC3, PC4, PC5, PC6, PC7, PC8, and PC9 showed low correlations (all loading values ≤ 0.5) with nine principal components between different experimental groups.

**Figure 5:**
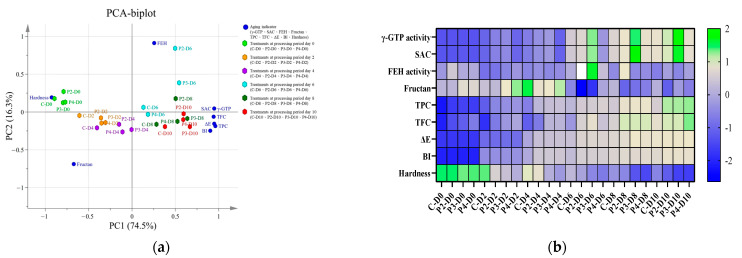
(**a**) Principal component analysis and (**b**) heat map analysis of functional compounds of garlic aging with different treatments. C: control; P: PEF pretreatment; D: heat treatment time of garlic aging. The number after P indicates the electric field strength (kV/cm) of the PEF pretreatment, and the number after D indicates the time (day) of garlic aging. The green color represents high expression and the blue color represents low expression in the heat map.

The factors obtained by principal component analysis were plotted into a biplot shown in Figure 5a. The PEF pretreatments and garlic aging process can be divided into four quadrants according to different indicators for garlic’s main components or indicators. The first quadrant represents hardness, while the second quadrant encompasses FEH activity, γ-GTP activity, and SAC content. The third quadrant represents fructan content, and the fourth quadrant includes TFC, ΔE, TPC, and BI. The 2 and 3 kV/cm PEF pretreatments had high correlations with γ-GTP activity and SAC content during garlic aging, while the 3 kV/cm PEF pretreatment positively promoted browning and TPC and TFC accumulations. However, the control and 4 kV/ cm PEF-pretreated groups were less relevant for SAC generation. Moreover, SAC content, TPC, TFC, and browning had significant changes after the fourth day of garlic aging. In addition, the fructan content was the main component change in the early stage of garlic aging, regardless of whether PEF was involved. Figure 5b further confirms that the formation of SAC was mainly P3-D8 and P3-D10, showing that the 3 kV/cm PEF pretreatment significantly impacted the SAC content. Additionally, the 2, 3, and 4 kV/cm PEF pretreatments were beneficial for the formation of TPC and TFC. However, the impacts of the PEF pretreatments on fructan content and browning were relatively insignificant.

Figure 5b also demonstrates the benefits of PEF-assisted garlic aging. After PEF treatment, the key indicator component SAC increase surpasses the control group under the same aging period. This result indicates that PEF treatment can enhance functional components in a shorter time. The aging process of garlic traditionally requires significant energy and time for heating. In contrast, PEF treatment consumes less energy and time, reducing the overall time and energy consumption in the aging process. Thus, this aligns with the United Nations Sustainable Development Goal (SDG) 12, “Responsible Consumption and Production”, contributing to the food industry’s development trends.

## 4. Conclusions

In this study, PEF pretreatment at 2 and 3 kV/cm showed a significant increase in SAC, TPC, and TFC generation during garlic aging caused by the disruption of garlic tissue and increased membrane permeability. However, PEF at 4 kV/cm resulted in membrane permeability and inhibited the γ-GTP activity and SAC content. Although it remains unclear whether PEF promotes browning through the conversion of organic sulfides or the browning of polyphenol oxidase, this study showed that PEF treatment can promote garlic browning and increase the polyphenol content. Moreover, the PEF pretreatments did not inhibit the FEH activity but significantly promoted it on day 6 during aging. Subsequently, the fructan content in aged garlic decreased, leading to texture softening. Principal component analysis showed that the PEF treatments positively impacted SAC content, TPC, and TFC in aged garlic but did not negatively impact browning or texture. In summary, PEF pretreatment shortens the aging time and increases the yield of SAC compounds and antioxidant substances (TPC and TFC). This study can potentially advance the application of PEF pretreatment in accelerating the aging process of fermented foods and enhancing the bioactive compounds of functional foods through PEF parameter optimization.

## Figures and Tables

**Table 1:** Eigenvalue, variability, and cumulative value for principal component analysis of functional compounds or indicators of different experimental groups.

	PC1	PC2	PC3	PC4	PC5	PC6	PC7	PC8	PC9
γ-GTP	0.951	0.049	−0.255	−0.117	0.111	0.001	0.030	0.003	−0.037
SAC	0.948	0.047	−0.271	−0.115	0.107	0.007	0.016	0.000	0.037
FEH	0.257	0.911	0.299	0.052	0.097	0.029	0.042	0.001	0.001
Fructan	−0.668	−0.687	0.192	0.048	0.197	0.037	0.043	0.002	0.001
TPC	0.962	−0.186	0.168	0.028	−0.021	−0.054	0.009	0.084	0.002
TFC	0.940	−0.061	0.032	0.305	0.096	−0.055	−0.075	−0.030	−0.001
ΔE	0.947	−0.155	0.189	−0.154	−0.016	0.111	−0.081	0.001	−0.002
BI	0.904	−0.245	0.272	−0.193	−0.053	−0.068	0.047	−0.051	0.001
Hardness	−0.929	0.193	0.071	−0.263	0.116	−0.075	−0.076	0.010	0.000
Eigenvalue	6.700	1.470	0.408	0.256	0.098	0.031	0.025	0.011	0.003
Variability (%)	74.47	16.30	4.53	2.84	1.09	0.34	0.28	0.12	0.03
Cumulative value (%)	74.47	90.77	95.30	98.14	99.23	99.57	99.85	99.97	100.00

## Data Availability

Data will be made available upon request.
